# Edaravone reduces oxidative stress and intestinal cell apoptosis after burn through up-regulating miR-320 expression

**DOI:** 10.1186/s10020-019-0122-1

**Published:** 2019-12-11

**Authors:** Jiaxiang Ke, Xi Bian, Hu Liu, Bei Li, Ran Huo

**Affiliations:** 1 Burn and Plastic Section, Qingdao Municipal Hospital Affiliated to Shandong University, Qingdao, China; 2 Burn and Plastic Section, Shandong Province Hospital Affiliated to Shandong University, Jiaozhou Road, Shibei District, Qingdao, 266011 Shandong Province China

**Keywords:** Edaravone, Oxidative stress, Apoptosis, MiR-320, Intestinal mucosa damage

## Abstract

**Background:**

Intestinal mucosa barrier dysfunction after burn injury is an important factor for causing mortality of burn patients. The current study established a burn model in rats and used a free radical scavenger edaravone (ED) to treat the rats, so as to investigate the effect of edaravone on intestinal mucosa barrier after burn injury.

**Methods:**

Anesthetized rats were subjected to 40% total body surface area water burn immediately, followed by treatment with ED, scrambled antagomir, or antagomiR-320. Intestinal mucosa damage was observed by hematoxylin-eosin staining and graded by colon mucosal damage index (CMDI) score. The contents of total sulfhydryl (TSH), superoxide dismutase (SOD), catalase (CAT) and malondialdehyde (MDA) were determined by spectrophotometry. Cell apoptosis, protein relative expression,and the in situ expressions of p-Akt and p-Bad were detected by flow cytometry, Western blotting and immunohistochemistry, respectively. The miR-320 expression was determined by quantitative real-time polymerase chain reaction.

**Results:**

ED alleviated intestinal mucosal damage caused by burn injury, down-regulated the levels of MDA, cytochrome C, cleaved caspase-9 and cleaved caspase-3, but up-regulated the levels of TSH, SOD, CAT and Bcl-2. We also found that ED could reduce oxidative stress, inhibit cell apoptosis, increase the expressions of p-Akt, p-Bad and miR-320, and decrease PTEN expression. PTEN was predicted to be the target gene for miR-320, and cell apoptosis could be promoted by inhibiting miR-320 expression.

**Conclusion:**

ED regulates Akt/Bad/Caspase signaling cascade to reduce apoptosis and oxidative stress through up-regulating miR-320 expression and down-regulating PTEN expression, thus protecting the intestinal mucosal barrier of rats from burn injury.

## Background

Physiological barrier injury caused by intestinal damage after burn injury is an important factor for causing the mortality of burn patients (Grimes et al., 2016; Ng et al., 2016). Severe burn injury, increases intestinal microvascular permeability increases, damages intestinal cell necrosis, apoptosis and intestinal mucosal barrier function impair, lead to displacement of intestinal bacterial and toxin, induction of systemic inflammatory response syndrome and multiple organ dysfunction syndromes (Zhang et al., 2017a; Osuka et al., 2017)*.* Recent studies showed that hypoxic ischemia and reperfusion injury in intestinal tissue after burn injury is possibly a main contributing to intestinal barrier damage (Miranda et al., 2018; Zhang et al., 2017b; Zhou et al., 2015; Tassopoulos et al., 2017). Therefore, protecting the function of intestinal mucosal barrier after burn injury could be effective to the prevention and treatment of intestinal infection and multiple organ dysfunctions.

Edaravone (ED) is a free radical scavenger that protects the cerebral functions, and is a first-line drug in clinical treatment of cerebral infarction (Parikh et al., 2017; Tokumaru et al., 2018). ED scavenges free radicals, inhibits lipid peroxidation and alleviates ischemia-reperfusion injury (Fujisawa & Yamamoto, 2016; Uchiyama et al., 2015)*,* moreover, as it has antioxidant properties, ED is the first free radical scavenger clinically proved to be a neuroprotective agent in Japan since 2001 (Minnelli et al., 2019)*,* and it plays an important role in alleviating oxidative stress in some diseases (Kikuchi et al., 2012)*.* Takeo Koizumi et al. (Koizumi et al., 2006) showed that ED could reduce the free radical precursors and their metabolites in extensively burned rats. The current study established a burn model in rats, which were treated by ED to explore the effects of ED on intestinal mucosa of after burn injury. The findings in the current study provide experimental basis for finding effective measures to the protection of intestinal barrier function after burn injury.

## Methods

### Animals and burn model

Wistar rats (male and female, weighting 180–220 g, aged 7 weeks old) were purchased from the Experimental Animal Center of Southern Medical University (http://portal.smu.edu.cn/sydwzx/info/1006/1075.htm). A total of 60 rats were housed in a room at 19–25 °C in 30–70% humidity under a 12-h light/dark cycle, and free access to food and water was provided to the rats. All experiments performed in the present study were approved by the Animal Ethics Committee of Qingdao Municipal Hospital Affiliated to Shandong University.

Twenty four hours (h) prior to the experiment, back hair of the rats were shaved, and the rats were fasted for 12 h before the experiment. A total of 60 rats were divided into 5 groups, namely, sham group (*n* = 10), model group (*n* = 10), ED group (edaravone, *n* = 20), scrambled group (*n* = 10) and antago group (*n* = 10). After the anesthesia, the rats were anesthetized by intraperitoneal injection of sodium pentobarbital (50 mg/kg), those in sham group were exposed to water at 25 °C for 15 s, while those in model group, ED group, scrambled group and antago group were exposed to boiled water at 100 °C for 15 s to create the burn model with 40% total body surface area (TBSA).

Immediately after the establishment of the burn injury, the rats were intraperitoneally injected with 2 ml Lactated Ringer solution’s (LRS). No special drugs were used in sham group or model group, while those in ED group, scrambled group and antago group were given 9 mg/kg ED (Mitsubishi Pharma Corporation, Japan) through intraperitoneal injection. In addition to injection of ED, the rats in scrambled group were given 2 μg scrambled antagomiR, while those in antago group were treated by 2 μg antagomiR-320. After the burn injury and treatment, the rats were housed in separate cages and given analgesic treatment with sodium pentobarbital (50 mg/kg). 24 h after establishment of burn injury, all the rats were euthanized by dislocating cervical vertebra after anesthesia via intraperitoneal injection of sodium pentobarbital (50 mg/kg). After laparotomy, small intestines of the rats were removed and parts of the intestines were stored in liquid nitrogen, while the rest was maintained in 10% formalin solution for subsequent experiments. The dosages of all animal drugs were calculated according to the equivalent dose conversion coefficient of human and animal drugs (Reagan-Shaw et al., 2008).

### Intestinal mucosal injury index analysis

The intestines of rats were rinsed in normal saline to observe the damages on intestinal mucosa, and damage indexes were graded by CMDI score as follows: 0 point represents no injury to the intestinal mucosa; 1 point represents that the surface of intestinal mucosa is smooth, no erosion or ulcer, but with mild hyperemia and edema; 2 points represents that the intestinal mucosa has congestion and edema, the mucosa is coarse and granular, with erosion or intestinal adhesion; 3 points represents necrosis and ulcers appeared on the surface of intestinal mucosa, which also has high congestion and edema (the maximum longitudinal diameter of the ulcer is shorter than 1.0 cm), moreover, the intestinal wall surface has necrosis and inflammation or the hyperplasia of intestinal wall; 4 points represents the maximum longitudinal diameter of ulcer is longer than 1.0 cm, or with total intestinal wall necrosis more severe than 3 points.

### Hematoxylin-eosin (HE) staining

The intestine mucosal tissues were fixed by 10% formalin solution and embedded in paraffin section. The samples were cut into 5 μm thick sections, and then stained by hematoxylin for 5 to 15 min (Beyotime Biotechnology, Shanghai, China) and then stained by eosin for 1 to 3 min (Beyotime Biotechnology, Shanghai, China). The tissue slices were observed under an optical microscope (BH-2, Olympus, Japan).

### Spectrophotometry

The intestinal mucosal tissue homogenate was prepared and centrifuged at 2000×*g* for 10 min, and the levels of sulfhydryl (TSH), superoxide dismutase (SOD), catalase (CAT), and malondialdehyde (MDA) in the supernatant were determined according to the manufacturer’s protocols of Total mercapto (−SH) measurement kit, SOD typed assay kit, CAT assay kit (Visible light), and MDA assay kit (all purchased from Jiancheng Bioengineering Institute, Nanjing, China). The absorbance values were measured using a visible spectrophotometer (N732, INSTRUNENT, Shanghai, China). TSH and CAT were detected at wavelengths of 405 nm, while MDA was detected at 532 nm, and SOD was detected at 550 nm.

### Apoptosis assay

The apoptosis assay was performed using Annexin V Apoptosis Detection Kit (KeyGen, China). The single cell suspension of intestinal cells was prepared by enzyme digestion. The cells were washed twice using phosphate-buffered saline (PBS), re-suspended in Annexin V binding buffer, and the Annexin V-FITC and propidium iodide (PI) buffer were then added to the cells and incubated together in the dark at 4 °C for 10 min. Cell apoptosis was determined by flow cytometry (Epics-XLII, Beckman, USA).

### Immunohistochemistry (IHC) assay

The sections (5 μm) were incubated with 3% H_2_O_2_ for 10 min, and then blocked by 5% goat serum (Zsbio, Beijing, China) for 15 min. Anti-p-Akt (rabbit, 1:200, #9271, Cell Signaling Technology, USA) and anti-p-Bad (rabbit, 1:200, ab28825, Abcam) antibodies were added to the sections and incubated overnight at 4 °C. After washing the sections by PBS for three times, the sections were incubated with goat anti-rabbit secondary antibody (goat, 1:2000, ab205718, Abcam) for 20 min at 37 °C. DAB detection kit (Beyotime Biotechnology, China) was used to visualize the sections, which were then observed under an optical microscope (BH-2, Olympus, Japan).

### Target gene prediction

Potential target gene for miR-320, and the binding sites of miR-125b and its target gene were predicted by Targetscan 7.2 (http://www.targetscan.org/).

### Western blotting

The small intestine mucosal tissues were used for Western blotting. The tissues were cut to sections and lysed by RIPA lysis buffer and protease inhibitor on ice for 10 min. The lysates were centrifuged at 12000×*g* for 20 min, and the supernatant was mixed with 5× loading buffer. 15 μl proteins were separate on 10% SDS-PAGE, and then transferred onto PVDF membranes. The protein concentrations were determined by BCA Protein Assay kit (Thermo Scientific, USA). The membranes were blocked by 5% milk powder overnight at 4 °C and incubated with primary antibodies (anti-p-Akt (rabbit, 1:200, #9271, Cell Signaling Technology, USA), anti-p-Bad (rabbit, 1:200, ab28825, Abcam), Bcl-2 (rabbit, 1:500, ab59348, Abcam), anti-Cytochrome C (mouse, 1:2000, ab13575, Abcam), anti-Cleaved Caspase-9 (C caspase-9, rabbit, 1:2000, ab2324, Abcam), anti-Cleaved Caspase-3 (C caspase-3 rabbit, 1:2000, ab2302, Abcam), anti-GAPDH (mouse, 1:2000, ab8245, Abcam), anti-PTEN (rabbit, 1:2000, #9552, Cell Signaling Technology, USA), anti-Akt (rabbit, 1:1000, #9272, Cell Signaling Technology, USA) and anti-Bad (rabbit, 1:2000, ab32445, Abcam)) for 1 h. Next, goat anti-rabbit secondary antibody (12,000, ab205719, Abcam) or goat anti-mouse secondary antibody (12,000, ab205718, Abcam) was used to further incubate the membranes for 40 min at room temperature. GAPDH served as an internal control. The protein bands were detected by SuperSignal West Dura Extended Duration Substrate (Pierce, USA) and X-ray Film (Kodak, USA), and analyzed by BandScan 5.0 system (Bio-Rad, Hercules, USA).

### Quantitative real-time polymerase chain reaction (qRT-PCR)

The small intestine mucosal tissues were cut to sections and total RNAs were extracted using TRIzol reagent (Invitrogen, USA) according to the manufacturer’s protocol. The purity and concentration of RNAs were determined by Nano Drop 2000 (Thermo, Scientific, USA), and cDNAs (2 μg) were synthesized by PrimeScript RT Master Mix kit (Takara, China). QRT-PCR was conducted in ABI PRISM 7500 real-time PCR (Applied Biosystems, USA) by SYBR PCR Master Mix (Applied Biosystems, USA), and the PCR cycles were set as follows: pretreatment at 95 °C for 30 s, followed by 40 cycles at 95 °C for 5 s, at 60 °C for 30 s, finally at 70 °C for 30 s, and preserved at 4 °C. Gene relative expressions were calculated by 2^-ΔΔCT^. The primer sequences used were listed in Table 1. U6 served as the endogenous control.

**Table 1:** The qRT-PCR primer sequences

Name	Forward primer: 5′-3’	Reverse primer: 5′-3’
miR-320	*AAAAGCTGGGTTGAGAGGG*	TGCGTGTCGTGGAGTC

### Statistical analysis

The data were analyzed by SPSS 17.0 statistical analysis software (SPSS, USA) and shown as mean ± standard deviation (SD). One-way analysis of variance (ANOVA) and student’s t test were conducted for the comparisons among groups. A *p* < 0.05 was considered to be a statistically significant difference.

## Results

### ED attenuated the intestinal mucosal tissue injury in burned rats

**Fig. 1:**
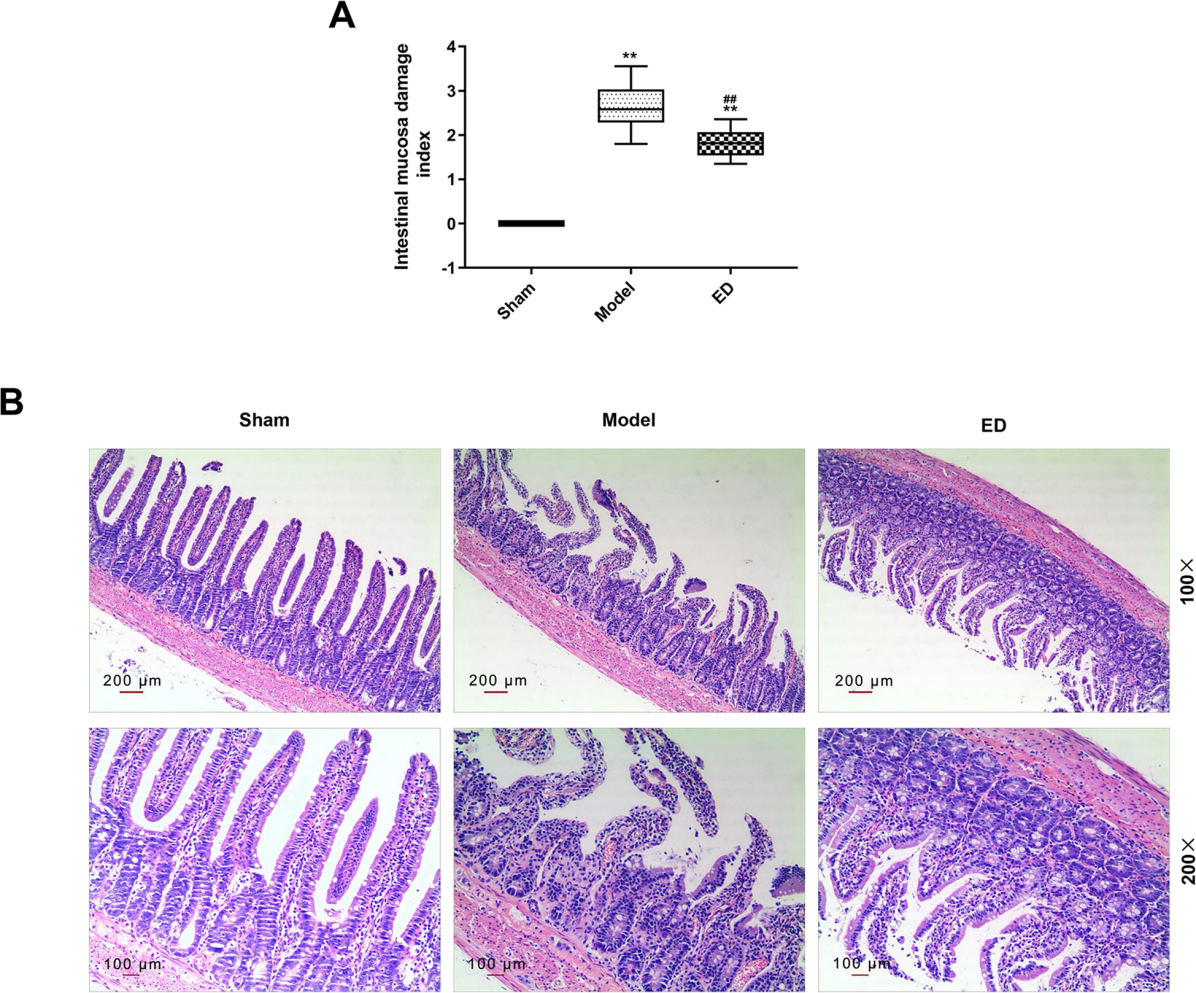
The change of intestinal mucosal tissue of rats after burn injury was observed. **a** The intestinal mucosa damage index was measured by colon mucosal damage index. **b** The pathological change of intestinal mucosal tissue was observed by using hematoxylin-eosin staining. A total of 60 Wistar rats (half male and half female) were divided into 5 groups, namely, sham group (25 °C water, *n* = 10), model group (100 °C water, *n* = 10), ED group (edaravone, 100 °C water, *n* = 20), scrambled group (100 °C water, *n* = 10) and antago group (100 °C water, *n* = 10). The rats were exposed to 25 °C or 100 °C water for 15 s after anesthesia (intraperitoneal injection of 50 mg/kg sodium pentobarbital). The ED group, scrambled group and antago group were given intraperitoneal injection of 9 mg/kg ED. Then the scrambled group was given intraperitoneal injection of 2 μg scrambled antagomir, and antago group was given intraperitoneal injection of 2 μg antagomiR-320. All rats were anesthetized and euthanized. ^**^P < 0.01 vs. Sham, ^##^*P* < 0.01 vs. Model

Thirty rats were divided into 3 groups, namely, sham group, model group and ED group. The rats in model group and ED group were exposed to water at 100 °C to construct the burn model, and then corresponding drugs were injected. All rats were euthanized, their small intestines were removed to observe the damage of intestinal mucosa, and the indexes of intestinal mucosa damage were graded by CMDI score. As shown in Fig. 1a, the intestinal mucosa damage indexes in model group and ED group increased as compared with sham group, while the index of the ED group decreased as compared with model group (*P* < 0.05).

The HE staining results (Fig. 1b) showed a clear structure of intestinal villi and mucosa and intact epithelial cells in the sham group, however, in the model group, the intestinal villi were arranged in disorder and some structures were completely destroyed, the epithelial cells were degenerated and necrotic, moreover, a large amount of necrotic and exfoliated tissues and mucus were observed in the intestinal cavity of the rats. Compared with model group, the above pathological changes were alleviated in the ED group, in which, the intestinal mucosa was orderly arranged but with complete structures, the epithelial cells were slightly necrotic, and a small number of cells were found in the intestinal cavity. These above results revealed that the intestinal mucosa of rats was damaged after burn injury, however, ED could alleviate the damage degree of intestinal mucosa.

### ED enhanced the antioxidant ability of intestinal mucosa in burned rats

**Fig. 2:**
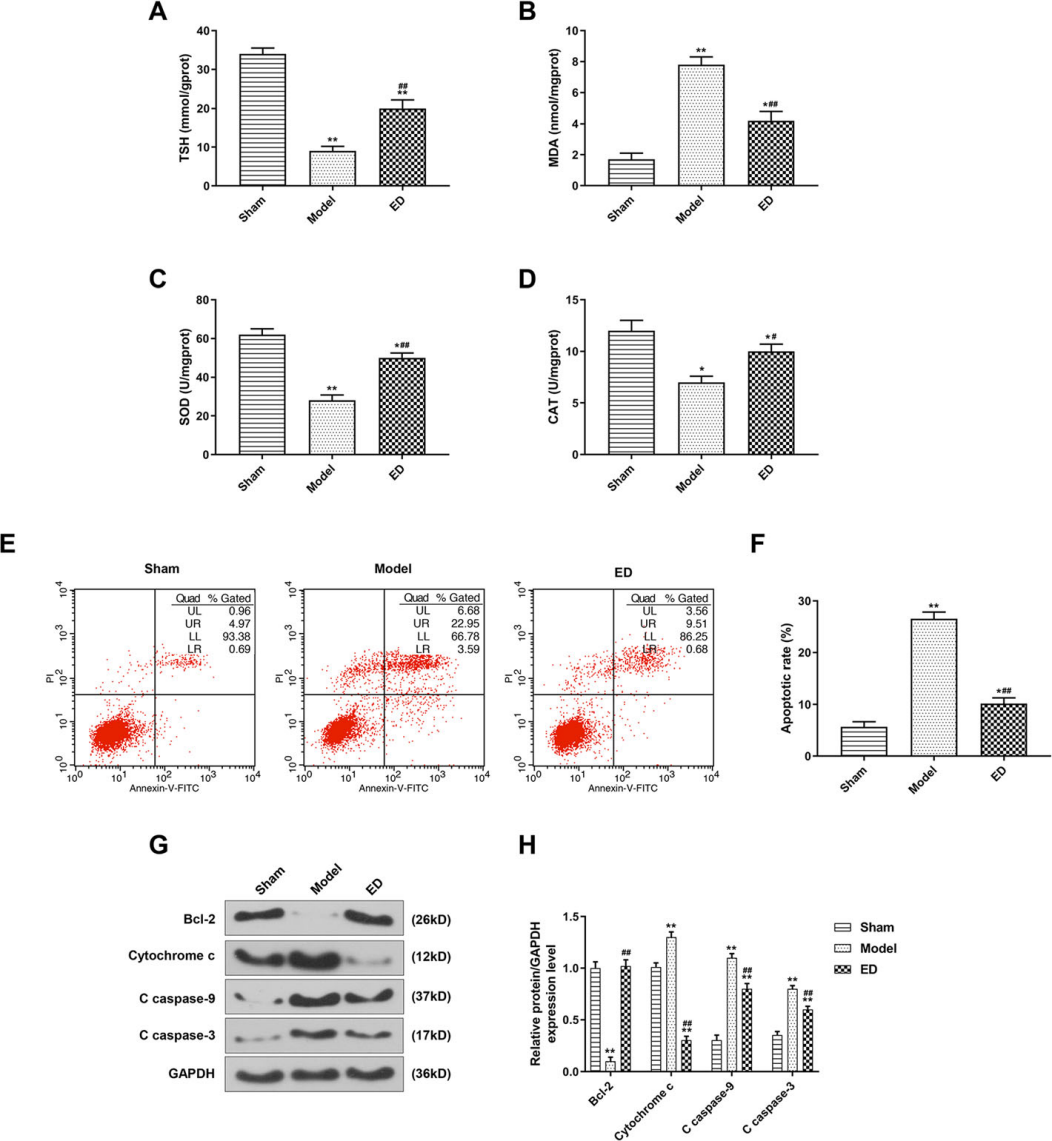
Oxidative stress and apoptosis were observed in intestinal tissue of burn model rat. **a**: The total sulfhydryl (TSH) expression was detected by spectrophotometry. The malondialdehyde (MDA) expression was detected by spectrophotometry. **b**: The malondialdehyde (MDA) expression was detected by spectrophotometry. **c**: The superoxide dismutase (SOD) expression was detected by spectrophotometry. **d**: The catalase (CAT) expression was detected by spectrophotometry. **e** The apoptosis was detected by flow cytometry. **f**: ED inhibited the apoptosis caused by burn injury. **g**: The Bcl-2, cytochrome c, C caspase-9 and C caspase-3 expressions were detected by Western blotting. **h**: ED decreased cytochrome c, C caspase-9 and C caspase-3 expressions and increased the Bcl-2 expression. ^*^P < 0.05 vs. Sham, ^**^P < 0.01 vs. Sham, ^##^*P* < 0.01 vs. Model

The contents of TSH and oxidative stress-related enzymes in intestinal mucosa of rats were determined by spectrophotometry. After burn injury, the levels of TSH, SOD and CAT were reduced, but MDA level was increased in model group compared with sham group. Moreover, the levels of TSH, SOD and CAT were reduced and MDA level was increased by ED treatment group compared with sham group, whereas the MDA level was reduced significantly in ED treatment group compared with model group (Fig 2a, b, c and d, *P* < 0.05), suggesting that oxidative stress damage occurred in the intestinal mucosa of the rats, whose antioxidant ability decreased after burn injury. Moreover, ED increased the levels of TSH, SOD and CAT, but reduced the level of MDA after burn injury, indicating that ED could alleviate oxidative damage in intestinal mucosa of rats after burn injury.

### ED inhibited cell apoptosis

The intestinal cell apoptosis was determined by flow cytometry, and the expressions of apoptosis-related proteins were detected by Western blotting. The data found that cell apoptosis rates increased in the model group and ED group compared with sham group, whereas ED markedly reduced the cell apoptosis rate after burn injury (Fig. 2f, *P* < 0.05). In addition, the Bcl-2 level in model group was lower than that in sham group, however, the levels of cytochrome C, C caspase-9 and C caspase-3 were higher in the model group (Fig. 2h, *P* < 0.05). Furthermore, compared with model group, the Bcl-2 level in ED group was up-regulated, whereas the levels of cytochrome C, C caspase-9 and C caspase-3 were down-regulated (Fig. 2h, *P* < 0.05), suggesting that the ED treatment inhibited cell apoptosis.

### ED up-regulated the levels of p-Akt, p-bad and miR-320

**Fig. 3:**
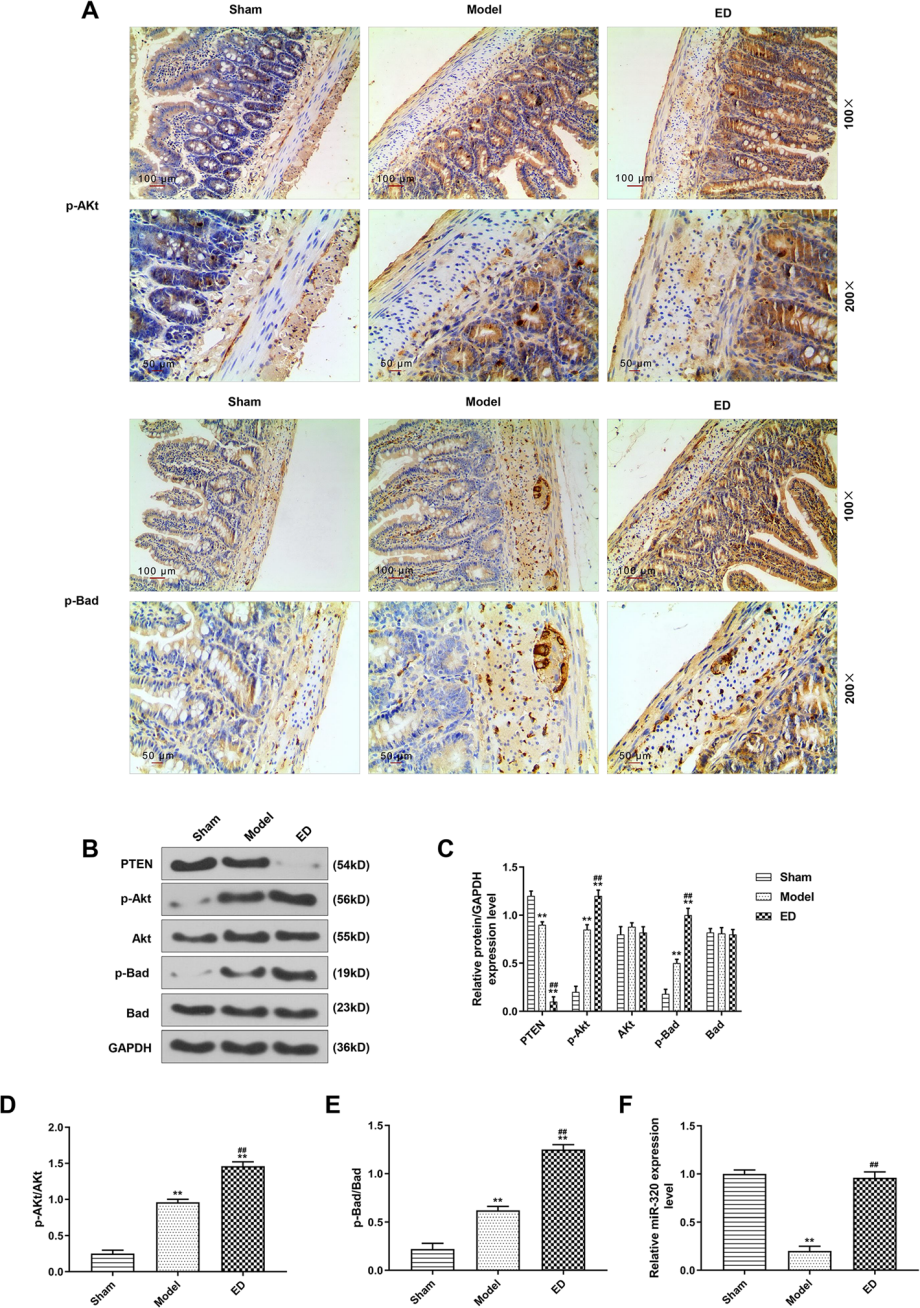
The p-Akt, p-Bad and miR-320 expressions were observed in different groups. **a**: The p-Akt and p-Bad were determined by immunohistochemistry assay. **b**: The expressions of PTEN, p-Akt, p-Bad, Akt and Bad were detected by Western blotting. **c**: ED decreased PTEN expression but increased p-Akt and p-Bad expressions. **d**: The p-Akt/Akt level in different groups. **e**: The p-Bad/Bad level in different groups. F: The miR-320 expression was determined by quantitative real-time polymerase chain reaction (qRT-PCR). ^**^P < 0.01 vs. Sham, ^##^*P* < 0.01 vs. Model

The expressions of p-Akt and p-Bad were determined by IHC assay and Western blotting, as shown in Fig 3a and b, the expressions of p-Akt and p-Bad in model group were higher than those in sham group, and the expressions of p-Akt and p-Bad in ED group increased compared with model group (*P* < 0.05), moreover, the results of western blotting also showed similar changes (Fig. 3c). We also found that compared with sham group, the expressions of p-Akt and p-Bad in model group and ED group were increased, whereas the expression of PTEN was decreased (Fig. 3c, *P* < 0.05). Furthermore, the expressions of p-Akt and p-Bad were higher, but the PTEN expression was lower in ED group than those in model group (Fig. 3c, *P* < 0.05), however, the expressions of p-Akt/Akt and p-Bad/Bad were higher in in model group than those in sham group, and higher in ED group than those in model group (Fig 3d and e, *P* < 0.05). In addition, the level of miR-320 was detected by qRT-PCR, and we found that miR-320 level was lower in model group than that in sham group, butit was up-regulated in ED group compared with model group (Fig. 3f, *P* < 0.05). These above results suggested that ED increased the levels of p-Akt and p-Bad, maintained the activities of Akt and Bad and reversed the inhibitory effect of burn injury on miR-320.

### Low expression of miR-320 promoted apoptosis

**Fig. 4:**
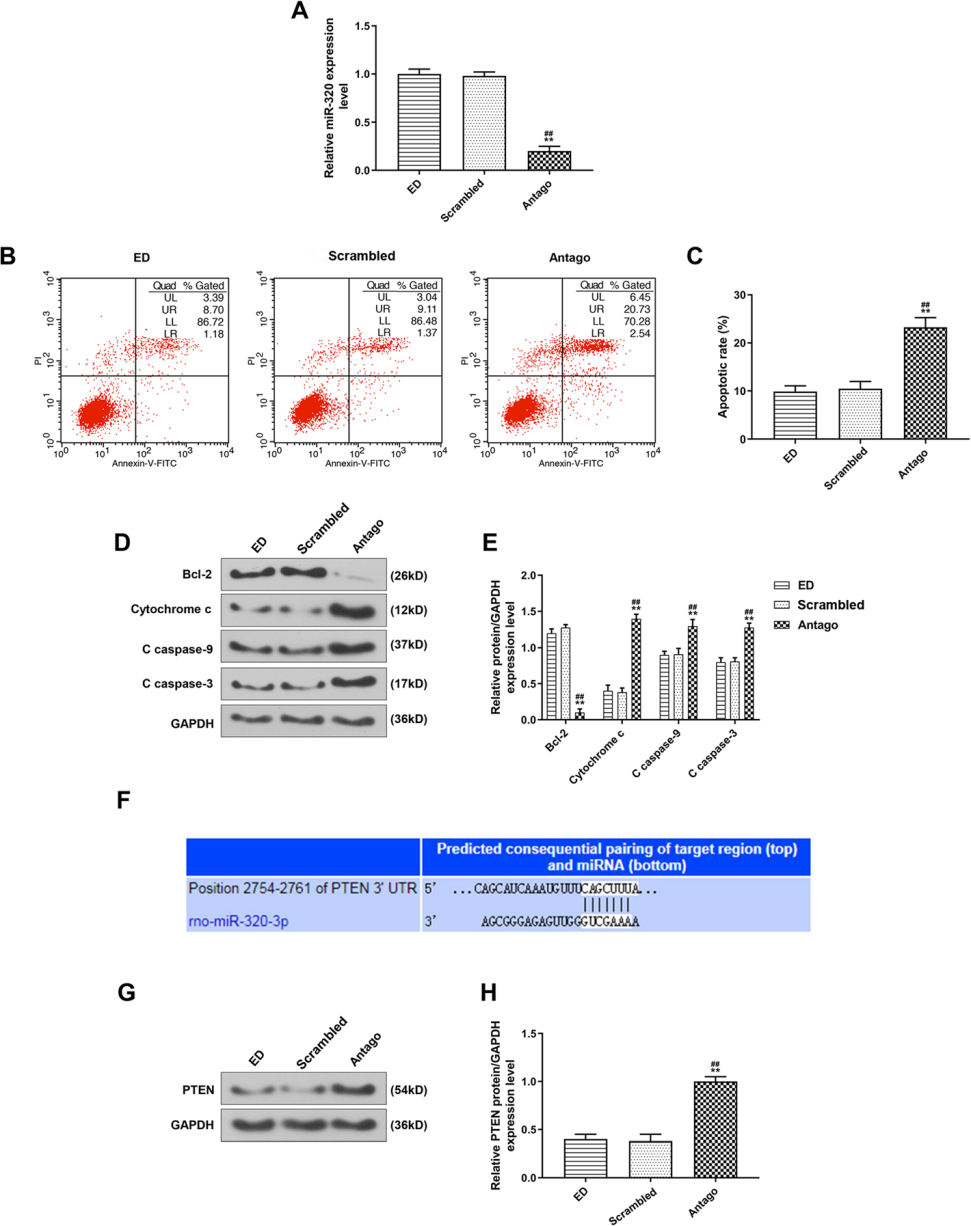
The apoptosis and the target gene of miR-320 were determined. **a**: The miR-320 expression was determined by qRT-PCR. **b**: The apoptosis was detected by flow cytometry. **c**: The apoptosis was up-regulated after inhibited miR-320 expressions. **d**: The Bcl-2, cytochrome c, C caspase-9 and C caspase-3 expressions were detected by Western blotting. **e**: After the inhibition of miR-320 expression, the expressions of cytochrome c, C caspase-9 and C caspase-3 were increased and the expression of Bcl-2 was decreased. **f**: The miR-320 potential target gene was predicted by Targetscan 7.2. **g**: The PTEN expression was detected by Western blotting. **h**: The PTEN expression in antago group was up-regulated. ^**^P < 0.01 vs. ED, ^##^*P* < 0.01 vs. Scrambled

Thirty mice were divided into 3 groups, namely, ED group, scrambled group and antago group. The mice were exposed to water at 100 °C to construct the burn model and injected with corresponding drugs. All the rats were euthanized and their small intestines were removed for subsequent experiments. The level of miR-320 in different groups was detected by qRT-PCR, and we found that the miR-320 level was down-regulated in antago group compared with that in ED group or scrambled group (Fig. 4a, *P* < 0.05). Then, the apoptosis rate and expressions of apoptosis-related proteins were determined by flow cytometry and Western blotting. Compared with ED and scrambled groups, we discovered that the apoptosis rate was increased and the levels of cytochrome C, C caspase-9 and C caspase-3 were up-regulated in antago group, but the Bcl-2 level was down-regulated (Fig. 4 c, e, *P* < 0.05), suggesting that the low expression of miR-320 up-regulated the expressions of pro-apoptotic proteins, down-regulated the protein expression of bcl-2, and promoted cell apoptosis.

### PTEN was the target gene for miR-320

Targetscan 7.2 predicted that PTEN was the target gene for miR-320. Then, the level of PTEN was detected by Western blotting, and the data found that the level of PTEN was significantly up-regulated in antago group (Fig. 4h, *P* < 0.05). Thus, PTEN was confirmed to be the target gene of miR-320.

## Discussion

Burn injury will change intestinal structures, functions and damage intestinal mucosal barrier, thus leading to intestinal bacterial displacement and severe burn infection (Earley et al., 2015; Cannon et al., 2016)*.* Therefore, it is of great clinical significance to protect the integrity of intestinal structure and promote the recovery of intestinal mucosa from burn injury. The current study showed that the CMDI scores of the rats in the burn model increased, moreover, HE staining observed disordered intestinal villi, degeneration and necrosis of intestinal cells, and the intestinal mucosa of the burn rats was damaged. However, ED could effectively reduce the damage indexes of rat intestinal mucosa, alleviate the changes of intestinal mucosa structure, showing significant protective effects on intestinal mucosa.

The damage of intestinal mucosal barrier caused by burn is related to the intestinal oxidative stress injury and lipid peroxidation (Yalcin et al., 2012; Xu et al., 2014; Cheng et al., 2017)*.* MDA can reflect the degree of lipid peroxidation in the body and damage degree of cells under oxidative stress (Martinez Aranzales et al., 2015)*.* SOD and CAT are common enzymes in the endogenous antioxidant system, and can eliminate excessive reactive oxygen species (ROS) in the body and be consumed in large quantities under increased oxidative stress (Bhattacharyya et al., 2014; Tasanarong et al., 2014). In vivo*,* sulfhydryl (−SH) is a main participant in cell antioxidant system, and the content of TSH indirectly reflects the ability of the organism to scavenge oxygen free radicals (Naghii, 2002)*.* ED is an oxygen-free radical scavenger that scavenges free radicals and reduces lipid peroxidation of cell membrane, thus preventing cell peroxidation damage (Ahmadinejad et al., 2017)*.* The current study found that ED could effectively reduce the MDA level and increase the levels of TSH, SOD and CAT. These data suggested that ED protects the intestinal mucosa by restoring the activity of antioxidant enzymes, improving the antioxidant capacity of intestinal mucosa and reducing oxidative stress.

The balance between the proliferation and apoptosis of intestinal cells is essential in maintaining the function of intestinal mucosal barrier (Assimakopoulos et al., 2013; Cao et al., 2018)*.* Studies showed that the oxidative stress caused by burn injury will increase the production of oxygen free radical, cause lipid peroxidation and eventually induce apoptosis in the body (Guo et al., 2015; Nielson et al., 2017)*.* Our study observed an increase of intestinal cells apoptosis in the burned rats, which was similar to previous studies. The occurrence of apoptosis is regulated by apoptosis-related genes, including Bcl-2, Bax, caspase family and Fas membrane proteins, etc. (Hristova et al., 2018)*.* Bcl-2 is an anti-apoptosis protein and has the function of inhibiting the release of apoptosis factors such as cytochrome C (Negroni et al., 2015)*.* Cytochrome C and the caspase family play an important role in the process of apoptosis, and cytochrome C can interact with caspase-9 to activate the downstream effector enzyme caspase-3. Cleaved caspase-3 (C caspase-3), which is the main effector for cell apoptosis, could cause cell apoptosis (Guo et al., 2015; Zhou et al., 2017)*.* In this study, the reduced apoptosis was observed after ED treatment and the Bcl-2 level was up-regulated, however, the levels of apoptotic factors were down-regulated. Therefore, we concluded that ED could regulate the apoptosis of intestinal cells by increasing the expression of Bcl-2 and decreasing the expressions of pro-apoptotic factors.

Akt plays a key role in the PI3K/Akt pathway and is involved in the regulation of cell survival and apoptosis (Lv et al., 2018; Rana et al., 2015). Activating Akt (p-Akt) can inhibit the expressions of pro-apoptotic proteins such as Bax and caspase-9, increase the anti-apoptotic protein expression and promote cell survival (Hu et al., 2017)*.* p-Akt can not only phosphorylate pro-apoptotic protein Bad (p-Bad) and block the combination of Bad with anti-apoptotic protein Bcl-xl/Bcl-2, but also prevent the release of cytochrome C from mitochondria into the cytoplasmic and the interaction of cytochrome C with caspase-9, so as to form an apoptosis complex to activate caspase-3 (Liu et al., 2015)*.* Activity of Akt is negatively regulated by PTEN in cell apoptosis, adhesion and some other biological processes, and absence of PTEN can activate Akt (Li et al., 2015)*.* The current study observed that ED promoted the phosphorylation of Akt and Bad and inhibited PTEN expression, suggesting that the protective effect of ED on intestinal mucosal barrier may be achieved by regulating Akt signal to inhibit the apoptosis of intestinal cells in the burned rat.

MicroRNAs (miRNAs) are short non-coding RNA sequences that play critical roles in a variety of physiological and pathological processes (Fromm et al., 2015). Burn injury, disease and some other factors could change the expressions of miRNAs and therefore negatively affect intestinal homeostasis (Morris et al., 2017). MiR-320 is a member of the miRNAs (Song et al., 2014), to date, evidence demonstrated that miR-320 was involved in a variety of pathological processes, including myocardial ischemia reperfusion injury, and it is associated with cell proliferation, apoptosis and death (Zhang et al., 2016)*.* In the current research, we observed an increase in miR-320 expression after ED treatment, and found that PTEN was the target gene for miR-320. Moreover, the PTEN expression and apoptosis were increased after antagomir-320 was used to inhibit miR-320. MiRNAs can regulate various life activities by binding to 3′-untranslated region (3′-UTR) of their target genes (Park & Shin, 2014). These above results suggested that the ED could inhibit cell apoptosis by up-regulating miR-320 expression, therefore inhibiting PTEN expression and phosphorylating Akt and Bad.

## Conclusion

In conclusion, the present study confirmed the protective effect of ED on intestinal mucosal barrier in rats after burn injury, and such an effect of ED was achieved by up-regulating the expression of miR-320 to inhibit PTEN expression and by regulating Akt/Bad/Caspase signaling cascade to reduce apoptosis and oxidative stress. Thus, ED has a great clinical potential to be explored as a therapeutic drug for treating burn patients. However, limitation still exists in the study, as we did not study the effects of different dosages of ED for pharmacological study.

## Data Availability

The analyzed data sets generated during the study are available from the corresponding author on reasonable request.

## Abbreviations

ANOVA: Analysis of variance
CAT: Catalase
*ED*: *Edaravone*
HE: Hematoxylin-eosin
IHC: Immunohistochemistry
LRS: Lactated Ringer solution's
MDA: Malondialdehyde
PBS: Phosphate-buffered saline
PI: Propidium iodide
qRT-PCR: Quantitative real-time polymerase chain reaction
*ROS*: *Reactive oxygen species*
SD: Standard deviation
SOD: Superoxide dismutase
TBSA: Total body surface area
TSH: Total sulfhydryl
